# Prevent Safety Threats in New Construction through Integration of Simulation and FMEA

**DOI:** 10.1097/pq9.0000000000000189

**Published:** 2019-06-24

**Authors:** Nora Colman, Kimberly Stone, Jennifer Arnold, Cara Doughty, Jennifer Reid, Sarah Younker, Kiran B. Hebbar

**Affiliations:** From the * Department of Pediatrics, Division of Pediatric Critical Care, Children’s Healthcare of Atlanta, Atlanta, Ga.; † Department of Pediatrics, Division of Emergency Medicine, Seattle Children’s Hospital and University of Washington School of Medicine, Seattle, Wash.; ‡ Department of Pediatrics, Maternal, Fetal, Neonatal Institute, Johns Hopkins All Children’s Hospital, St. Petersburg, Fla.; § Section of Emergency Medicine, Department of Pediatrics, Baylor College of Medicine, Texas Children’s Hospital, Houston, Tex.; ¶ Department of Pediatrics, Children’s Healthcare of Atlanta, Atlanta, Ga.

## Abstract

**Introduction:**

The *built environment* may impact safety and decisions made during the design phases can have unintended downstream effects that lead to patient harm. These flaws within the system are latent safety threats (LSTs). Simulation-based clinical systems testing (SbCST) provides a clinical context to examine the environment for safety threats postconstruction. Integration of Failure Mode Effect Analysis (FMEA) with SbCST provides a framework to identify, categorize, and prioritize LSTs before patient exposure.

**Methods:**

We implemented SbCST in a newly constructed pediatric subspecialty outpatient center before opening. We used in-situ simulations to evaluate both routine and high-risk clinical scenarios pertinent to each clinical area. FMEA was used as a methodology to assign risk, prioritize, and categorize LSTs identified during the simulation.

**Results:**

Over 3 months, we conducted 31 simulated scenarios for 15 distinct subspecialty clinics involving 150 participants and 151 observers. We identified a total of 334 LSTs from 15 distinct clinics. LSTs were further classified into process/workflow, facility, resource, or clinical performance issues.

**Conclusions:**

Integration of SbCST and FMEA risk assessment is effective in evaluating a new space for safety threats, workflow, and process inefficiencies in the postconstruction environment, providing a framework for prioritizing issues with the greatest risk for harm.

## Abstract 2

Supplemental Digital Content is available in the text.

## INTRODUCTION

Due to the increasing demands on healthcare systems to grow and expand, new construction and remodeling are common in modern day hospitals.^1^ Studies demonstrate that the *built environment*, which is the physical space that supports patient care and interacts with healthcare personnel, patients, equipment, and technology to impact safety.^1^ Decisions made during the design phase can have significant unintended, downstream effects that can lead to patient harm. These flaws within the system, or accidents waiting to happen, are known as latent safety threats (LSTs).^2,3^ Latent conditions may be difficult to detect proactively and may contribute to adverse events if not remediated with safety barriers.^1^ Despite the interaction of the built environment on safety, there is a need for a formalized way to more efficiently evaluate a newly built space for LSTs before opening for patient care.^4^

Simulation-based clinical systems testing (SbCST) provides a context to examine the process, environment, and human factors^5,6^ for a newly built environment in the postconstruction, preoccupancy phase of development. However, currently, there is a lack of a standard methodology by which to categorize and prioritize the LSTs identified during simulation testing.^4,7^

Failure mode and effects analysis (FMEA) is a proactive risk assessment tool endorsed by the Agency for Healthcare Research and Quality (AHRQ) and Institute for Healthcare Improvement, and is used by a multidisciplinary team to evaluate a process.^8–10^ Use of FMEA meets accreditation requirements set by The Joint Commission, which requires healthcare systems to proactively identify and remediate flaws in a system or processes that could put patients at risk for adverse events.^8,9,11^ The FMEA risk assessment process guides stakeholders to review, evaluate, and record failure modes, and identify cause and effect relationships to correct the failure before harm to a patient or staff occurs.^8,11^ The standard FMEA process requires teams to create a process map by “imagining” possible LSTs. SbCST and FMEA used synergistically is a novel strategy that enhances the impact that either of these methodologies would have when applied alone.

Combining SbCST and FMEA provides a unique opportunity for environmental evaluation and prioritization not possible with traditional FMEAs. Rather than imagining potential LSTs, teams simulate a process in its entirety, actively experiencing, witnessing, and assessing patient care through a platform that can bring to light the unpredictability of interacting systems and human factors to more effectively detect LSTs.^2^

In this article, we describe our experience with integrating SbCST and FMEA in the postconstruction, preoccupancy phase of design to identify and prioritize potential safety threats before opening an outpatient subspecialty clinic.

## METHODS

This study was a prospective simulation-based investigation that occurred over 3 months. Thirty-one simulation scenarios were conducted for 15 distinct subspecialty clinics to probe the environment for LSTs and process/workflow inefficiencies. We complete an FMEA for each clinical area immediately following each SbCST event. A final report describing each LST was categorized and prioritized by severity and distributed to the scoring team.

### Setting

Children’s Healthcare of Atlanta’s Center for Advanced Pediatrics is a state of the art, newly constructed 6-story, 260,000-square-foot facility serving over 30 clinical subspecialties, providing radiographic and laboratory services and estimated to see 100,000 patient visits in the first year. The architectural design of this building occurred over a 9-month time frame. We spent 12 months evaluating processes and workflows and 3 months conducting SbCST. The last SbCST event was conducted one month before the facility opening.

### Simulations

SbCST participants included frontline staff and physicians, who worked in existing clinics and were relocated to the Center for Advanced Pediatrics building. Additional participants included community emergency response teams, family volunteers, and embedded participants who played the role of the patient or parent. Individual staff members were recruited by clinical leaders in each area to participate. Administrative, operational, and clinical stakeholders such as the chief executive, nursing, medical officer, practice director, nursing director, physician division directors, and representatives from quality improvement and accreditation participated in the development, implementation, and evaluation phases of SbCST. Although administrative and operational leaders may not routinely participate in daily patient care, there was some overlap in physician leaders who participated as both stakeholders and participants during SbCST.

To guide scenario development, a team of clinical, administrative, and operational leaders performed a needs assessment to define key priorities for testing. The needs assessment process included brainstorming sessions, review of available process maps, and in-person interviews. The simulation team derived scenario content from these priorities and aligned the clinical context (simulated scenario) with SbCST goals to test multiple objectives. For example, one routine nephrology clinic visit simulation evaluated multiple objectives, including patient check-in, urine sampling, blood pressure measurement, and patient discharge. The process of simulation scenario design was similar to that of process mapping, where all processes of scenario progression were outlined and detailed.^12,13^

Simulated scenarios represented both routine situations encountered with high frequency and low frequency, high-risk scenarios pertinent to the patient clinic population (**Appendix A**, **Supplemental Digital Content 1**, http://links.lww.com/PQ9/A107). Although keying in on unique processes to each clinic, scenarios were also aimed at evaluating key AHRQ safe hospital design principles: standardization, staff fatigue/efficiency, reduction in communication breakdown, control/eliminate sources of infection, the role of automation, adjacencies, and patient and family involvement in care.^1,14^

We conducted individual testing sessions for one subspecialty clinic at a time. Each simulation block lasted approximately 4–5 hours and included registration and prebriefing. The prebrief for each session lasted 45 minutes and included a discussion of confidentiality, goals, and objectives of the scenario, and orientation to the mannequin and new clinical space. Each session included 1–3 distinct scenarios, lasting 20 minutes. Although participants actively probed the environment by performing clinical tasks related to patient care, observers documented and took note of any patient threats, issues with equipment, and workflow, or process inefficiencies using a standardized questionnaire based on the AHRQ safe hospital design principles. Observers also recorded participant comments regarding inefficiencies or challenges they experienced during the simulation. We placed observers in predetermined strategic locations where processes relevant to their expertise would be performed. Observations and recordings by the observers were discussed during debriefing.

### Debriefings

The simulation and scripted debrief was used to conduct the risk analysis. A structured 30-minute debriefing followed each simulation scenario. One hour was allotted to score FMEA findings for the entire simulation block. Debriefings included a scripted PowerPoint introduction and were led by facilitators trained in healthcare debriefing techniques and FMEA methodology.^15^ By applying simulation-based debriefing techniques, facilitators probed participants to explore further how the built environment and physical space impacted their workflow or threatened safety. During the debriefings, participants and observers identified any LSTs pertinent to each scenario. Each LST was then further explored to identify what effect would result if the threat occurred. A member of the simulation team transcribed all comments into a preformatted FMEA template during the discussion (**Appendix B**, **Supplemental Digital Content 2**, http://links.lww.com/PQ9/A108).

### The FMEA Process

We modeled the FMEA scoring tool off of the FMEA template used by Children’s Healthcare of Atlanta’s Quality and Patient Safety Department. This tool applied a 4-point Likert scale for each category of severity, occurrence, and detection. Immediately following the debrief, stakeholders participated in the FMEA scoring process where each LST was assigned a severity, occurrence, and detection score. Stakeholders were provided with a scoring rubric with anchoring descriptors (Table 1).^10^ Consistent with traditional FMEA scoring, stakeholders discussed each failure mode and potential impacts. Numerical scores were assigned based on group consensus determined through group discussion.^16–18^ The simulation team functioned as an impartial party to facilitate discussion and scoring, but stakeholders determined the final score as experts in their clinical area.

**Table 1.**
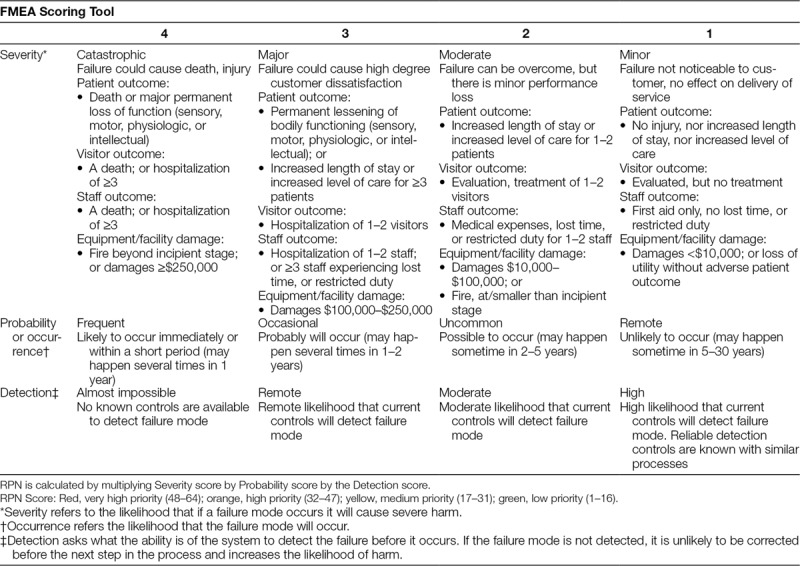
Failure Mode and Effect Analysis Scoring System

Once the group agreed on the severity, occurrence, and detection ranking, the team calculated a risk priority number (RPN) by multiplying [severity × occurrence × detection],^10^ with equal weight given to each component. The RPN score was then further classified into very high (RPN 48–64), high (RPN 32–47), medium (RPN 17–31), or low priority (RPN 1–16) (Table 1).

### Issue Categorization

We further categorized each potential LST into a resource, process/workflow, facility, or clinical performance LST. Resource LSTs were related to personnel, medication, and equipment that were either missing, malfunctioned, or unable to use. Process/workflow LSTs were related to policies or procedures that did not work and anticipated in the clinical setting. Facility LSTs referred to facility or space concerns that were not conducive to effective, efficient, and safe patient care. Clinical performance LSTs referred to gaps in knowledge, technical skills, or institutional processes that could be the focus of future simulation-based training. A final FMEA report that categorized and prioritized each threat was distributed to stakeholders.^9^ We prioritized the LSTs with the highest RPN score (those with potential to result in patient harm) as opportunities for improvement (OFI) that required immediate attention and possible corrective action before the clinic opening. Accountability and oversight of change implementation were left in the hands of quality and operational leadership.

## RESULTS

**Fig. 1.**
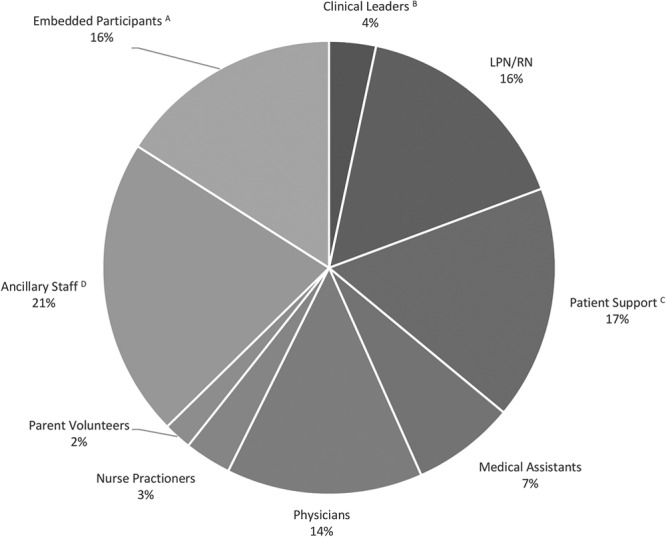
Participant demographics. ^A^Embedded participants include embedded patients, parents, community emergency and fire response, and critical care transport. ^B^Clinical leaders include nurse educators and assistant nurse managers. ^C^Patient support staff includes registration and patient access. ^D^Ancillary staff includes physical therapists, nutritionists, speech therapists, occupational therapists, respiratory therapists, phlebotomists, and technicians.

SbCST included 150 participants and 151 observers (Fig. 1). A total of 334 LSTs from 15 distinct clinics was identified (Table 2). Thirty-six very high priority LSTs, with a high likelihood for patient harm, including possible death, were prioritized as OFIs that needed corrective actions to be remediated by stakeholders before clinic opening.

**Table 2.**
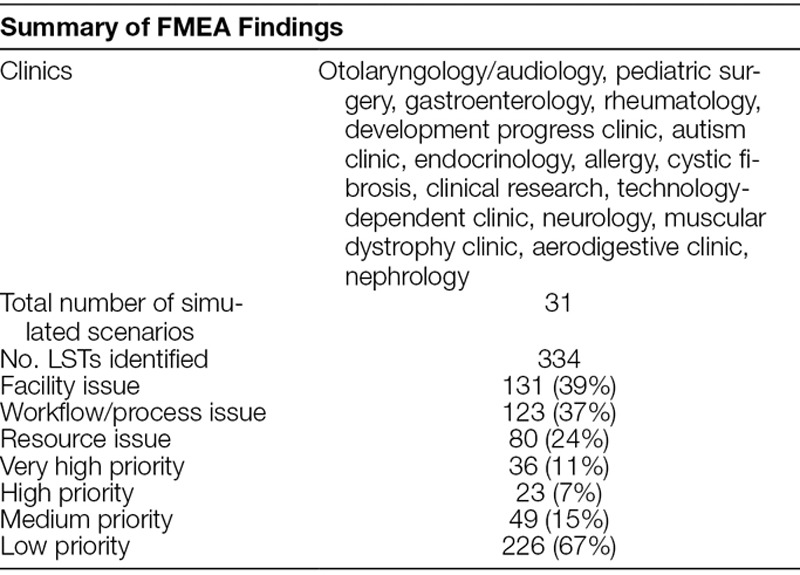
Summary of Latent Safety Threats Identified during FMEA Risk Assessment Analysis

Twenty-six LSTs, including 7 very high priority threats, were common to multiple clinical areas (Table 3). Very high priority threats included processes surrounding emergency preparedness and notification processes, the proximity of antibacterial hand sanitizer to clinic rooms, location of the sharps disposal container, infection control regarding the movement of cystic fibrosis patients throughout the building, accessibility of resuscitation bags, and impact of building climate on testing reagents. Lower priority LSTs included physician use of tracking boards, signage and wayfinding, transportation of laboratory specimens, and orientation of examination tables in the rooms. Stakeholders discussed low priority LSTs that did not require corrective actions before clinic opening.

**Table 3.**
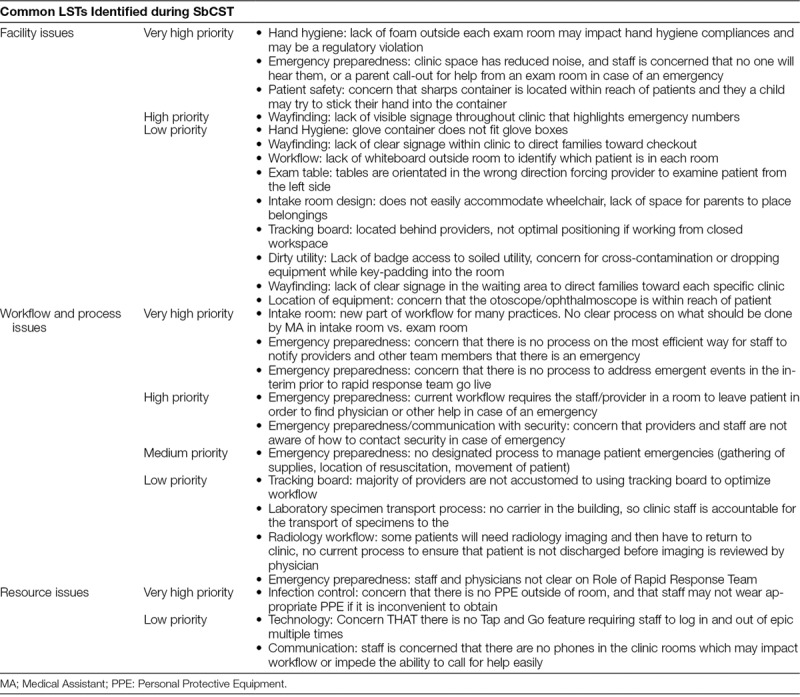
Common LSTs Identified in Multiple Subspecialty Areas

## DISCUSSION

We describe an innovative approach that integrates SbCST with FMEA to identify and prioritize LSTs in the postconstruction phase of design. Ours is the first project to describe the integration of these two methodologies.

Design teams are often unfamiliar with how the built environment impacts safety and even less familiar with ways to prevent these flaws from being incorporated into the final design.^19^

Studies by Adler^20^, Geis^21^, and Ventre et al^22^ used simulation as a vehicle to evaluate new healthcare facilities for LSTts. These studies support the fact that LSTs are inherent in new design and suggest that simulation can be incorporated into the post-occupancy evaluation process to mitigate risk. Results of these studies yielded a descriptive report of unprioritized findings.^20–22^ Additionally, Davis et al^8^ applied simulation and FMEA to evaluate processes related to obstetrics. However, neither of these studies describe the application of both simulation with FMEA as a method to evaluate a new environment. Simulation and FMEA together take observed threats and further categorize and prioritize them so that findings are more informative, less overwhelming, and can aid stakeholders in focusing their efforts to mitigate risk.

The integration of two methodologies, SbCST with FMEA risk assessment, allowed our multidisciplinary teams to interact with their built environment dynamically. Although Ashley and Armitage^18^ have challenged the validity and scoring process of FMEA, healthcare systems are driven to apply FMEA to meet accreditation requirements set by JHACO.^11^ This synergistic approach increases the yield of SbCST, particularly in terms of prioritizing LSTs, and provides a shared experiential component making FMEA less abstract. The simulation further minimizes the subjective nature of FMEA that comes with variation in the team’s experience, knowledge, and perceptions^23^ by providing stakeholder teams with the same experience around which to frame the team’s discussion and assign a risk priority score.^18^ Teams witnessed and experienced the failure mode happening instead of contemplating how it could happen.^24^ Furthermore, debriefings improve team consensus in scores by providing an avenue for individual rationales to be discussed by the entire multidisciplinary team, promoting team learning, understanding perspectives, and improving communication for which we could not account in mathematical procedures used to assess risk.^18^ Teams used this combined process to identify LSTs effectively with the opportunity to mitigate threats before the opening of our new facility. Through SbCST, we identified 334 LSTs, delineated which had the potential to result in patient harm, and aided leaders in prioritizing items that required immediate attention.

Common trends across clinical areas emerged, as multiple teams identified similar LSTs related to the impact of the new space on common existing processes and workflows. LSTs identified had the potential to harm patients or staff, resulting in delayed patient care, and impair workflow, and process efficiency. The impact of LSTs may further contribute to poor patient/family satisfaction, communication breakdown, increase infection risk, violation of accreditation policies, or increase financial costs to the system.^9^ Most common trends included LSTs related to low frequency but high-risk clinical scenarios around emergency preparedness. Despite previous contemplative process planning for the management of decompensating patients, participants identified new LSTs in the emergency notification system and resource acquisition. Simulation and re-creation of these low frequency yet high-risk events uncovered potential threats that were not realized in previous risk assessment exercises. Simulations highlighted that work as performed differed from how the planning teams conceptualized work as being done.^24^ This example highlights the complexity of patient care that cannot be realized in a static environment and illustrates the power that medical simulation has in integrating elements of human factors, process improvement, systems engineering, and healthcare science.

Many teams also recognized new LSTs in low risk yet frequently occurring clinical encounters. Process and workflow issues identified included flow of patients through the clinical space, use of electronic tracking boards, and issues with the discharge process. Many of these inefficiencies were a result of moving from a smaller floor plan to a larger physical space. Although the new space offered advantages, the larger footprint made transferring the previous staffing models and workflow challenging.

Design elements such as patient room size, size of intake rooms, and height of mounted foam dispensers, while considered the accepted standard by the architect team, did not meet the needs of the patient population being served. Simulation highlighted how the exam room size did not accommodate tracheostomy patients who were typically accompanied by multiple caregivers during clinic visits. The size and layout of intake rooms did not easily accommodate patients who use wheelchairs. Mounting of hand sanitizer, while meeting American Disability Act requirements (maximum height of 44 inches, a minimum height of 15 inches from the ground)^25^ was still at a height that children could potentially put hand sanitizer in their mouth, posing an unintended safety risk.

Sixty-one percent of the LSTs found were categorized as facility or resource LSTs, suggesting that despite exhaustive planning, certain elements of design did not interact with providers in an intended manner. Interaction with the environment through simulation highlighted how assumptions made with work as imagined did not translate into work as done.^24^ One-third of LSTs were categorized into process and workflow, resulting in significant changes made to improve workflow efficiency and enhance patient safety. One clinic completely overhauled their process for the flow of patients through their clinic. Other changes included installation of cooling unit in the clinical research lab to preserve samples; updated wayfinding signage; modification of the location of mounted foam dispensers and storage of personal protective equipment; updated registration staffing model; and change in the access to the dirty utility room from pin code to badge access.

FMEA exercises enhanced with SbCST are feasible as healthcare institutions have adopted simulation and recognized it as a critical way to measure and impact safety.^26–28^ The ability to predict how the built environment will impact safety is limited when healthcare personnel interacts with their built environment in ways that were not anticipated by the design team. Simulation can better model this complex integration and serve as a supplement to process mapping and risk assessment. Assessment of the built environment is dependent on a team of experts with an in-depth understanding of quality, patient safety, and clinical operations. Translating the use of FMEA from traditional risk assessment to application in SbCST is feasible as healthcare leaders, and stakeholders have familiarity with the process of describing LSTs and effects.

### Challenges and Limitations

Challenges of implementing SbCST and FMEA include logistics that require early inclusion of the simulation team in design planning, collaboration with quality and safety partners, and scheduling of large multidisciplinary meetings. A unified commitment to the activity, alignment of testing objectives with stakeholders, and adequate time for completion of SbCST and OFIs is essential for project success.

Communication with large numbers of participants via email alone can prove challenging. Face-to-face meetings with smaller groups helped foster engagement. The level of commitment and effort from the clinical teams directly impacts the thoroughness of the FMEA evaluation. Clear identification of testing objectives and specifically directed communications helped to promote engagement from stakeholders. Time to run simulations must be allotted postconstruction yet before opening for patient care. Inadequate time between SbCST and facility opening may make it difficult for changes to be made before the patient encounters.

Specific LSTs discovered in our testing may not be generalizable to other institutions as the clinical processes and workflows vary greatly amongst healthcare systems. Also, a high degree of simulation expertise and professional hours is necessary to conduct SbCST and variability in simulation resources may impact the feasibility of implementing this type of project. This type of work should be prioritized, as many studies have demonstrated that administrative planning is often not sufficient to predict the array of problems that arise when delivering patient care.^21,22,29^

## CONCLUDING SUMMARY

Integration of SbCST and FMEA risk assessment can be incorporated into the design evaluation process as a way to systematically evaluate a new space for safety threats, workflow, and process inefficiencies. This methodology further provides a framework for prioritizing issues with the greatest risk for harm. Disseminating this process amongst the simulation and quality communities could improve how healthcare facilities are designed and tested in the future. Further research and examination of data by those with knowledge in improvement science, operations, and human factors are necessary to determine the impact that SbCST has on new healthcare design and patient safety.

## ACKNOWLEDGMENTS

We acknowledge Ashley Dalpiaz, MSN, RN, and the Children’s Healthcare of Atlanta Simulation Center and the many simulation centers that have done work to pave the way for this publication. Specifically, Kelly Wallin, MSN, BSN, and the Texas Children’s Hospital’s simulation team. The simulation community’s knowledge sharing, sharing of success, and failures have played a role in the completion of this manuscript.

## DISCLOSURE

The authors have no financial interest to declare in relation to the content of this article. This study was performed at Children’s Healthcare of Atlanta, Emory University.

## Supplementary Material
