# Supplementary material for: Prevent Safety Threats in New Construction through Integration of Simulation and FMEA
Source: Pediatr Qual Saf. 2019 Jun 24;4(4):e189. doi: 10.1097/pq9.0000000000000189 (PMC6708643; doi:10.1097/pq9.0000000000000189)
Supplement: Supplementary file 1 [file pqs-4-e189-s001.docx]

| **Clinical Area** | **Scenario Description** |
| --- | --- |
| Aerodigestive Clinic | 1. Patient presents for routine visit at aerodigestive clinic. Patient requires imaging at radiology and is seen by pulmonary and ENT providers. Patient requires laryngoscopy by ENT/pulmonary which takes place in a procedure room. |
| Allergy Clinic | 1. Routine visit for allergy testing, patient develops anaphylaxis requiring albuterol and epinephrine. 2. Routine visit for allergy testing. Patient develops anaphylaxis requiring epinephrine but further decompensates and 911 is called. |
| Autism Clinic | 1. Routine clinic visit, patient seen for feeding evaluation and is seen by multiple consultants. 2. Food pantry specialist brings food from the parking lot up to the pantry and delivers a meal to a patient room. |
| Clinical Research | 1. Patient is seen for a routine clinic visit for a research study. Patient eats meal during clinic visit, labs are obtained, urine sample is obtained, and study drug is administered. 2. Patient is seen for a routine clinic visit for research study. EKG is obtained, patient’s port is accessed, and study drug is administered. |
| Cystic Fibrosis Clinic | 1. Patient seen for routine clinic visit requiring multidisciplinary consultants to see patient. Patient requires labs, imaging, and is transported to radiology. |
| Development Progress Clinic | 1. New patient evaluation who requires physical therapy, social work consultation, and provider examination. |
| Endocrinology Clinic | 1. Patient with known Type I Diabetes presents to clinic with concern for DKA and requires transfer to the emergency department. 2. Adolescent patient with Type I Diabetes presents with hypoglycemia requiring juice and glucagon. 3. Adolescent patient with known Congenital Adrenal Hyperplasia requires Solucortef injection teaching. |
| Gastroenterology Clinic | 1. Infant seen for routine visit, needs gastric tube replacement and nutrition consult. 2. New adolescent CP patient requiring weight and wheelchair weight, requires nutrition consult, and nasogastric tube placement with radiologic confirmation. |
| Muscular Dystrophy Clinic | 1. Adolescent patient with muscular dystrophy who is wheelchair bounds presents for a routine clinical visit. Patient requires radiology visit, labs, PFT testing, and is seen by multiple subspecialists |
| Nephrology Clinic | 1. Routine nephrology visit, patient requires flu vaccine. 2. Routine nephrology visit, patient found to be hypertensive, provides urine sample for testing. |
| Neurology Clinic | 1. Adolescent patient is seen for routine neurology and EEG visit. 2. Patient seen for neuropsychiatric appointment. Patient has a seizure and requires emergency intervention and calling of 911. |
| Otolaryngology/Audiology Clinic | 1. Patient with laryngomalacia requires laryngoscopy in ENT clinic. 2. Patient comes into clinic without appointment in respiratory distress. Requires emergency management of respiratory decompensation. 3. Patient is seen as a routine visit for a failed hearing test. Patient requires Spanish interpreter for visit. |
| Pediatric Surgery Clinic | 1. Patient comes into clinic without appointment in significant respiratory distress with cyanosis, requiring airway management and resuscitation. 2. Adolescent female seen in clinic for uterine bleeding, requires Implantable Uterine Device placement 3. Patient with polydactyly seen in clinic for digit removal. Patient visit requires Spanish interpreter. |
| Rheumatology Clinic | 1. Patient with Juvenile Idiopathic Arthritis presents to clinic for joint injection. Patient receives sedation, develops respiratory depression requiring oxygen. Patient then returns to baseline and is discharged. 2. Patient with chronic recurrent osteomyelitis presents for routine clinic visit and requires x-rays. |
| Technology Dependent Clinic | 1. Patient is seen as a “new” patient evaluation who requires physical therapy, social work consultation, and provider examination. |

Appendix A. Summary of scenarios by clinical area
