# Supplementary material for: Prevent Safety Threats in New Construction through Integration of Simulation and FMEA
Source: Pediatr Qual Saf. 2019 Jun 24;4(4):e189. doi: 10.1097/pq9.0000000000000189 (PMC6708643; doi:10.1097/pq9.0000000000000189)
Supplement: Supplementary file 2 [file pqs-4-e189-s002.docx]

| **Failure Mode and Effect Analysis Worksheet** | | | | | | | | |
| --- | --- | --- | --- | --- | --- | --- | --- | --- |
| **Threat Detected** | **LST Category** | **Potential Failure Effect** | **Action Recommended by Scoring Team** | Severity | Occurrence | Detection | RPN | **30-day Post Simulation Action Taken** |
| *What is the process step, change or feature under investigation?* |  | *What is the impact on the customer if this failure is not prevented or corrected?* | *What are the recommended actions for reducing the occurrence of the cause or improving detection?* |  |  |  |  | *What action will be taken in relation to this finding and what will the follow-up plan be?* |
| *Resource issues: relate to personnel, medication, and equipment, whether missing, malfunctioning, or unable to use due to lack of provider familiarity with the device* | | | | | | | | |
|  | **Resource Issues** |  |  |  |  |  |  |  |
|  | **Resource Issues** |  |  |  |  |  |  |  |
| *Process/systems issues:* *relates to process, policies, or procedures that do not work as well as anticipated in the clinical setting* | | | | | | | | |
|  | **Process/Systems Issue** |  |  |  |  |  |  |  |
|  | **Process/Systems Issue** |  |  |  |  |  |  |  |
| *Facility issues:* *refer to facility or space set up concerns that are not conducive to effective, efficient, and safe patient care* | | | | | | | | |
|  | **Facility Issue** |  |  |  |  |  |  |  |
|  | **Facility Issue** |  |  |  |  |  |  |  |
| *Clinical performance issues:* *refer to* *cognitive skills, technical skills, institutional process, or knowledge of clinical personnel that can be a focus for future simulation-based training* | | | | | | | | |
|  | **Clinical Performance Issue** |  |  |  |  |  |  |  |

Appendix B. Example of Failure Mode and Effect Analysis Tool. Each failure mode can be further divided by resource issue, process or systems issues, facility issues, or clinical performance issues

LST-Latent safety threat, RPN-Risk Priority Number
